# Cardiac autonomic dysfunction in obese normotensive children and adolescents

**DOI:** 10.1590/0103-0582201432210213

**Published:** 2014-06

**Authors:** Isabelle Magalhães G. Freitas, Josiane Aparecida Miranda, Pedro Augusto C. Mira, Carla Marcia M. Lanna, Jorge Roberto P. Lima, Mateus Camaroti Laterza

**Affiliations:** 1 UFJF, Juiz de Fora, MG, Brasil

**Keywords:** obesity, arterial pressure, heart rate, autonomic nervous system, child, adolescent

## Abstract

**OBJECTIVE:**

To test the hypothesis that obese normotensive children and adolescents present impaired cardiac autonomic control compared to non-obese normotensive ones.

**METHODS:**

For this cross-sectional study, 66 children and adolescents were divided into the following groups: Obese (n=31, 12±3 years old) and Non-Obese (n=35, 13±3 years old). Obesity was defined as body mass index greater than the 95^th^ percentile for age and gender. Blood pressure was measured by oscillometric method after 15 minutes of rest in supine position. The heart rate was continuously registered during ten minutes in the supine position with spontaneous breathing. The cardiac autonomic control was assessed by heart rate variability, which was calculated from the five-minute minor variance of the signal. The derivations were the index that indicates the proportion of the number of times in which normal adjacent R-R intervals present differences *>*50 miliseconds (pNN50), for the time domain, and, for the spectral analysis, low (LF) and high frequency (HF) bands, besides the low and high frequencies ratio (LF/HF). The results were expressed as mean±standard deviation and compared by Student's t-test or Mann-Whitney's U-test.

**RESULTS:**

Systolic blood pressure (116±14 *versus* 114±13mmHg, p=0.693) and diastolic blood pressure (59±8 *versus* 60±11mmHg, p=0.458) were similar between the Obese and Non-Obese groups. The pNN50 index (29±21 *versus* 43±23, *p*=0.015) and HF band (54±20 *versus* 64±14 normalized units - n.u., *p*=0.023) were lower in the Obese Group. The LF band (46±20 *versus* 36±14 n.u., *p*=0.023) and LF/HF ratio (1.3±1.6 *versus* 0.7±0.4, *p*=0.044) were higher in Obese Group.

**CONCLUSIONS:**

Obese normotensive children and adolescents present impairment of cardiac autonomic control.

## Abstract (es)

**OBJETIVO:**

Probar la hipótesis de que niños y adolescentes obesos normotensos presentan disfunción autonómica cardiaca cuando comparados a individuos no obesos también normotensos.

**MÉTODOS:**

Estudio transversal con 66 niños y adolescentes, divididos en los grupos Obeso (n=31, 12±3 años) y No obesos (n=35, 13±3 años). Se definió la obesidad por el índice de masa corporal superior al percentil 95, considerándose edad y sexo. Se verificó la presión arterial clínica por oscilometría después de 15 minutos de reposo en posición supina. Se registró la frecuencia cardiaca durante 10 minutos en la posición supina, con respiración espontánea. Se evaluó el control autonómico cardiaco por la variabilidad de la frecuencia cardiaca, calculada a partir de los cinco minutos de menor variancia de la señal. Fueron derivados el índice que indica la proporción del número de veces en que los intervalos R-R normales sucesivos presentan diferencia de duración superior a 50 milisegundos (pNN50) para el dominio del tiempo y, para el análisis espectral, las bandas de baja (LF) y alta (HF) frecuencias, además de la razón entre las bandas espectrales de baja y alta frecuencia (LF/HF). Los resultados se presentaron como promedio±desviación estándar, siendo comparados por la prueba t de Student o por la prueba U de Mann-Whitney.

**RESULTADOS:**

Los niveles de presión arterial sistólica (116±14 versus 114±13mmHg, *p*=0,693) y diastólica (59±8 versus 60±11mmHg, *p*=0,458) fueron semejantes entre los grupos Obeso y No obeso, respectivamente. El índice pNN50 (29±21 versus 43±23; *p*=0,015) y la banda HF (54±20 versus 64±14 unidades normalizadas - u.n.; *p*=0,023) fueron menores en el Grupo Obeso. La banda LF (46±20 versus 36±14 u.n.; *p*=0,023) y la razón LF/HF (1,3±1,6 versus 0,7±0,4; *p*=0,44) fueron más grandes en el Grupo Obeso.

**CONCLUSIONES:**

Niños y adolescentes obesos, aunque normotensos, presentan perjuicio del control autonómico cardiaco.

## Introduction

Childhood obesity^(^ 1 ^)^ affects around 16% of the world population aged 6 to 19 years^(^ 2 ^)^. In Brazil, data from the Brazilian Institute of Geography and Statistics (IGBE - Instituto Brasileira de Geografia e Estatística) for 2010 shows that approximately 14% of children and 5% of adolescents were obese^(^ 3 ^)^. In addition to being highly prevalent, obesity in children and adolescents is responsible for the emergence and development of cardiovascular diseases^(^ 1 ^,^ 2 ^)^.

Over recent decades, results of indirect analysis of cardiac autonomic modulation of heart rate variability (HRV) have been found to have a direct associated with cardiovascular prognosis^(^ 4 ^)^. Findings show that the lower the HRV, the greater the chance of coronary events occurring^(^ 5 ^)^.

Additionally, dysfunctions of autonomic cardiac control have been described among the pathophysiologic characteristics of childhood obesity^(^ 6 ^-^ 8 ^)^. Among obese children and adolescents, reductions are observed in HRV and vagal modulation while sympathovagal balance increases^(^ 6 ^,^ 7 ^)^. These findings suggest, at least in part, that cardiac autonomic dysfunction in obese children and adolescents is related to sympathetic hyperactivation in detriment to vagal activation. 

However, in the majority of studies that have assessed HRV, the obese children and adolescents studied had significantly higher blood pressures than their non-obese peers^(^ 6 ^,^ 8 ^)^. This hemodynamic characteristic may itself be an independent cause of the cardiac autonomic dysfunction observed in this population. There is in fact a direct association between high arterial blood pressure levels and impairment of cardiac autonomic modulation^(^ 9 ^,^ 10 ^)^. In view of this, in order to attempt to exclude the effect of high arterial blood pressure on cardiac autonomic modulation, the objective of this study was to test the hypothesis that normotensive obese children and adolescents would exhibit impaired cardiac autonomic modulation when compared with normotensive non-obese individuals.

## Method

The sample size calculation was based on an article published previously^(^ 6 ^)^ and estimated that a minimum of 23 individuals in each group would be needed to achieve test power of 90% with an α error of 5%. A total of 31 normotensive obese children and adolescents were therefore recruited at the pediatric obesity and hypertension clinic run by the Fundação Imepen in Juiz de Fora, MG, Brazil, and 35 non-obese controls were recruited in the local community. All volunteers were aged 8 to 17 years, were normotensive and were not on any type of medication. 

Both volunteers and their legal representatives received explanations of all of the procedures involved in the study and, after both agreed to participation, free and informed consent forms were signed. This project was approved by the Research Ethics Committee at the Universidade Federal de Juiz de Fora (UFJF, protocol number 0051/2009) and was conducted at the UFJF University Hospital and Physical Education and Sports Department.

Since the inclusion criteria were obesity or healthy weight combined with normal blood pressure, the following procedures were used to define the participants: body mass and height were measured using a balance with built-in stadiometer (Filizola^(r))^ and body mass index (BMI) was calculated by dividing body weight in kilograms by the square of height in meters. Obesity was defined as BMI above the 95th percentile for age and sex^(^ 11 ^)^. Arterial blood pressure was measured in an upper limb after 15 minutes at rest in the supine position, noninvasively, using the auscultatory method with a mercury-column sphygmomanometer (Takaoka^(r))^, on two different days prior to the start of the experimental protocol. The choice of cuffs appropriate to arm circumference was made as recommended by the V Brazilian Directives on Arterial Hypertension (V Diretrizes Brasileiras de Hipertensão Arterial)^(^ 12 ^)^. Individuals were classed as normotensive if their means for two systolic and diastolic arterial blood pressure measurements were below the 90th percentile for age and sex^(^ 12 ^)^, which were 120mmHg for systolic pressure and 80mmHg for diastolic pressure. Individuals were excluded if they were on any type of medication and/or had a physician-diagnosed metabolic, cardiovascular or hormonal disease, in addition to obesity. These criteria were checked during clinical consultations conducted by the physician responsible for the clinic.

Physical activity was measured using the Habitual Physical Activity Questionnaire, which quantifies the number of minutes spent engaged in habitual physical activity during the 12 months preceding administration and has been validated for the juvenile Brazilian population^(^ 13 ^)^.

During the investigation, arterial blood pressure was measured non-invasively and automatically by the oscillometric method (Dixtal, DX 2020), with the cuff positioned on the volunteer's right upper limb. Heart rate was recorded continuously in the supine position using a heart rate monitor (Polar^(r)^, S810i). In order to evaluate autonomic cardiac control, data on the interval between each pair of heart beats (iRR) were sent to a microcomputer, by the pulse receptor's data transmission port to *Polar Precision Performance* ^(r)^ software, using an infrared signal interface. All signals used were within a maximum error of 3%. The data were then transferred to Matlab, version 6.0, for automatic selection of the five minutes of least variance, using a previously implemented routine^(^ 14 ^,^ 15 ^)^. This selection is made automatically using a moving window and the mathematical algorithm only analyzes the five minute period (uninterrupted) that exhibits the least variance. The sections selected were then analyzed visually and were only used for the HRV analysis if there were no obvious irregularities in the R-R intervals. These five-minute time series were then exported to *Kubios HRV Analysis*, version 2.0 software^(^ 15 ^)^. This application was used to correct artifacts, using the program's medium level filter, and to calculate the HRV indexes, for the time domain, and the mean R-R intervals (MNN), in milliseconds (ms), which represents the inverse of heart rate; the standard deviations for normal R-R intervals (SDNN), in ms, which reflects HRV; the square root of the mean of the squares of the differences between normal R-R intervals (RMSSD), in ms, and the percentage of adjacent R-R intervals with a difference greater than 50ms (pNN50), which reflects cardiac vagal modulation. To estimate the function for power spectrum density using the nonparametric Fourier rapid transform method^(^ 16 ^)^, the trend component was removed from the time series, using the *a priori* smoothing method^(^ 17 ^)^, after piecewise cubic spline interpolation, at a frequency of 4Hz. The spectral analysis of HRV was based on calculation of the power spectrum density for a low frequency band (LF - 0.04 to 0.15Hz), which reflects predominantly sympathetic modulation, and for a high frequency band (HF - 0.15 to 0.4Hz), which reflects cardiac vagal modulation, expressed both as absolute power (milliseconds squared - ms^2)^ and as normalized units (n.u.), and also the LF/HF ratio, which reflects sympathovagal balance.

All volunteers were assessed during the afternoon in order to avoid influence from the circadian cycle. The anthropometric measurements were taken first and then, after 15 minutes at rest in the supine position, arterial blood pressure was measured. Next, a 10-minute continuous recording of heart rate was made in order to calculate HRV.

Data were expressed as mean±standard deviation. The Kolmogorov-Smirnov test was used to verify normality of data. Possible differences between groups were analyzed using Student's *t* test for independent samples, where variables had normal distribution, or the Mann-Whitney U test if data distribution was not normal. Analysis of covariance (ANCOVA) was used to control for the possible effects of age and sex. Statistica, version 8.0 (Statsoft, USA) was used for all statistical tests and differences were considered significant when *p*<0.05.

## Results

Results for anthropometric and hemodynamic variables and habitual physical activity are shown in Table 1. Groups were similar in terms of age, habitual physical activity level and, as expected, there were no significant differences in systolic or diastolic arterial blood pressures. Additionally, blood pressures were within the range considered ideal. 

**Table 1:**
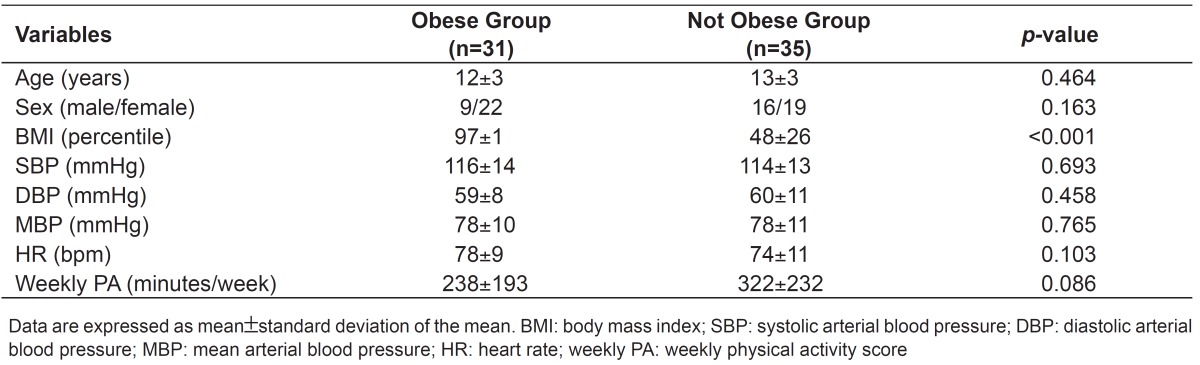
Anthropometric characteristics, hemodynamics at rest and weekly physical activity for Obese and Not Obese groups

The time-domain and frequency-domain results for HRV are shown in Table 2. The MNN, SDNN and RMSSD indexes were similar for both groups and the pNN50 index was lower in the Obese Group than in the Not Obese Group. With regard to frequency-domain HRV results, the spectral bands LF and HF were similar in both groups in terms of absolute power. However, the HF band (n.u.) was significantly lower and the variables LF (n.u.) and LF/HF were higher in the Obese Group. Among the variables used in the ANCOVA, it was observed that obesity was an independent factor for low values of pNN50 and the HF band (n.u.) and for high values for the LF band (n.u.) and the LF/HF ratio in the Obese Group (Table 3).

**Table 2:**
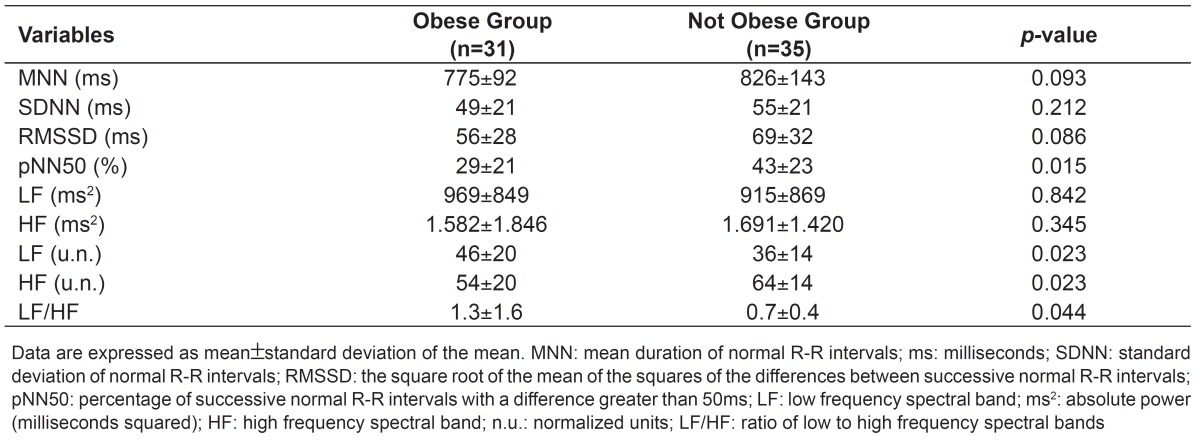
Variability of heart rate in time and frequency domains for Obese and Not Obese groups

**Table 3:**
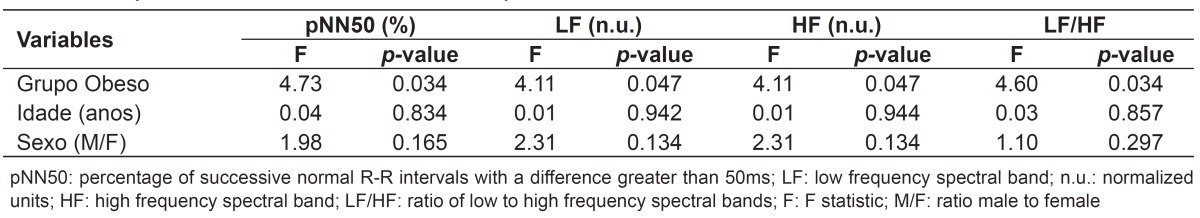
Analysis of covariance of heart rate variability

## Discussion

The primary finding of this study is that normotensive obese children and adolescents have abnormal cardiac autonomic modulation when compared with their non-obese, normotensive peers. The pNN50 index and HF band (n.u.), which indicate cardiac vagal modulation, were lower in obese individuals. Additionally, the LF band (n.u.), which reflects modulation that is predominantly sympathetic, and the LF/HF ratio, indicative of sympathovagal balance, were both elevated in the Obese Group. All of these results were independent of age and sex effects. 

The positive association between obesity and high blood pressure levels has consistently been reported^(^ 18 ^)^, and these hemodynamic changes have been primarily explained by hyperactivity of the sympathetic nervous system^(^ 19 ^)^. There is, therefore, clear evidence that excessive accumulation of body weight can provoke hemodynamic abnormalities and impairment of autonomic control^(^ 19 ^,^ 20 ^)^. For example, Guízar et al^(^ 8 ^)^ found that obese adolescents had significantly compromised sympathovagal balance, represented by an elevated LF/HF ratio. However, the adolescents investigated in that study, while normotensive, had significantly higher blood pressure levels than their non-obese peers. In the present study, there was also a negative change in sympathovagal balance manifest as higher LF/HF ratio among obese children and adolescents. However, in contrast with the research described by Guízar et al^(^ 8 ^)^, in which the obese subjects' blood pressure levels were already elevated, our results show that obesity was itself responsible for compromising the cardiac autonomic modulation of obese children and adolescents, irrespective of their resting blood pressure, since the blood pressure measurements for this sample did not only classify the individuals investigated as normotensive, but were also similar for the Obese Group and the Not Obese Group. 

There is no doubt that both compromised autonomic cardiac control and elevated arterial blood pressures are present in obesity. Riva et al^(^ 6 ^)^ conducted a study of obese adolescents who did not only have compromised sympathovagal balance, but also exhibited significantly reduced vagal modulation, represented by lower pNN50 index values. Therefore, if our results are considered side-by-side with those of these researchers^(^ 6 ^)^, it can be seen that the autonomic dysfunction observed in obesity may be characterized by sympathetic activation in detriment to vagal activation. Kauffman et al^(^ 21 ^)^ have published further evidence to support this, showing that obese adolescents had lower results for the HF band, converted into n.u., which is related to vagal modulation, while values for the LF band (also in n.u.) and the LF/HF ratio were elevated. Furthermore, these authors noted that the impairment of autonomic cardiac control seen in childhood obesity is associated with leptin levels, insulin resistance, and increased oxidative stress and inflammation, and that these relationships are primarily mediated by adipose tissue. 

The LF/HF ratio has been proposed as an accurate measure of sympathovagal balance of the heart, in that if this ratio is high it may indicate greater sympathetic modulation of the cardiovascular system^(^ 22 ^,^ 23 ^)^. Indeed, the normotensive Obese Group studied in the present research exhibited a higher LF/HF ratio than the Not Obese Group, which indicates that adipose tissue is one of the factors responsible for increased sympathetic stimulation, although other mechanisms may also be involved. Confirming this physiological representation of LF/HF, the spectral components of the LF and HF bands (in n.u.) show the balanced action of the two branches of the autonomic nervous system in control of heart beat^(^ 22 ^)^. The existence of a linear relationship has been shown between changes in the LF band (n.u.) and heart rate during tests involving incremental orthostatic postural maneuvers, reinforcing the theory that increases in the spectral values of this index indicate possible increases in sympathetic activation of the heart^(^ 24 ^)^. Thus, once again our results confirm the predominance of sympathetic cardiac modulation in the Obese Group, shown by significantly higher values for LF (n.u.), when compared with the Not Obese Group. As a consequence, the obese individuals studied exhibited impaired vagal cardiac activation, shown by their reduced HF (n.u.). 

Although they are not the object of study here, certain neurohumoral mechanisms could explain the cardiac autonomic dysfunction observed in obesity. The most important of these are increased levels of insulin, leptin, proinflammatory cytokines, oxidative stress and catecholamines^(^ 25 ^,^ 26 ^)^. These mechanisms are responsible for the sympathetic hyperactivation observed in obese individuals^(^ 19 ^)^ and, therefore, it can be speculated that the children and adolescents investigated here may also have disorders of these types. 

The detection of autonomic dysfunction in children and adolescents underscores the increased cardiovascular risk that is associated with childhood obesity, particularly the consequences of increased morbidity and mortality in adulthood^(^ 1 ^)^. These results are an alert to the need for actions designed to have an effect early on, avoiding progression to cardiovascular complications. Health education programs and interventionist measures to encourage lifestyle changes involving adoption of a balanced diet and regular physical activity should be conducted by multi-professional teams, in order to prevent and treat childhood obesity. 

One limitation of this study is that the maturity stages of these children and adolescents were not assessed. However, since it is known that maturity is influenced by both age and sex, an ANCOVA was conducted to control for the possible effects of these variables. Our results show that obese children and adolescents had lower results for pNN50 index and the HF band (in n.u.) and higher results for the LF band (in n.u.) and LF/HF ratio, irrespective of age and sex. Therefore, despite not having assessed maturity stage, it is improbable that this factor has skewed our results.

It can be concluded that even normotensive obese children and adolescents have impaired autonomic cardiac control when compared with their non-obese normotensive peers.
