# Supplementary material for: Cardiac autonomic dysfunction in obese normotensive children and adolescents
Source: Rev Paul Pediatr. 2014 Jun;32(2):244–9. doi: 10.1590/0103-0582201432210213 (PMC4183014; doi:10.1590/0103-0582201432210213)
Supplement: Translation

# Translation: Disfunção autonômica cardíaca em crianças e adolescentes obesos normotensos

**Authors:** Isabelle Magalhães G. Freitas, Josiane Aparecida Miranda, Pedro Augusto C. Mira, Carla Marcia M. Lanna, Jorge Roberto P. Lima, Mateus Camaroti Laterza

**Affiliations:** 1 UFJF, Juiz de Fora, MG, Brasil

**Keywords:** obesidade, pressão arterial, frequência cardíaca, sistema nervoso autônomo, criança, adolescente

## Abstract

**OBJETIVO:**

Testar a hipótese de que crianças e adolescentes obesos normotensos apresentam disfunção autonômica cardíaca quando comparados a indivíduos não obesos também normotensos.

**MÉTODOS:**

Estudo transversal com 66 crianças e adolescentes, divididos nos grupos Obeso (n=31, 12±3 anos) e Não Obeso (n=35, 13±3 anos). Definiu-se a obesidade pelo índice de massa corpórea maior que o percentil 95, considerando-se idade e sexo. Aferiu-se a pressão arterial clínica por oscilometria após 15 minutos de repouso em posição supina. Registrou-se a frequência cardíaca durante dez minutos na posição supina, com respiração espontânea. Avaliou-se o controle autonômico cardíaco pela variabilidade da frequência cardíaca, calculada a partir dos cinco minutos de menor variância do sinal. Foram derivados o índice que indica a proporção do número de vezes em que os intervalos R-R normais sucessivos apresentam diferença de duração superior a 50 milisegundos (pNN50) para o domínio do tempo e, para a análise espectral, as bandas de baixa (LF) e alta (HF) frequências, além da razão entre as bandas espectrais de baixa e alta frequência (LF/HF). Os resultados apresentam-se como média±desvio padrão, sendo comparados pelo teste *t* de Student ou pelo teste U de Mann-Whitney.

**RESULTADOS:**

Os níveis de pressão arterial sistólica (116±14 *versus* 114±13mmHg, *p*=0,693) e diastólica (59±8 *versus* 60±11mmHg, *p*=0,458) foram semelhantes entre os grupos Obeso e Não Obeso, respectivamente. O índice pNN50 (29±21 *versus* 43±23; *p*=0,015) e a banda HF (54±20 *versus* 64±14 unidades normalizadas - u.n.; *p*=0,023) foram menores no Grupo Obeso. A banda LF (46±20 *versus* 36±14 u.n.; *p*=0,023) e a razão LF/HF (1,3±1,6 *versus* 0,7±0,4; *p*=0,044) foram maiores no Grupo Obeso.

**CONCLUSÕES:**

Crianças e adolescentes obesos, mesmo normotensos, apresentam prejuízo do controle autonômico cardíaco.

## Introdução

A obesidade infantil^(^ ^1^ ^)^ acomete cerca de 16% da população mundial com idades de seis a 19 anos^(^ ^2^ ^)^. No Brasil, dados do Instituto Brasileiro de Geografia e Estatística de 2010 apontam que a obesidade atinge aproximadamente 14% das crianças e 5% dos adolescentes^(^ ^3^ ^)^. Além de ser altamente prevalente, a obesidade em crianças e adolescentes é responsável pelo surgimento e desenvolvimento de doenças cardiovasculares^(^ ^1^ ^,^ ^2^ ^)^.

Nas últimas décadas, os resultados obtidos pela análise indireta da modulação autonômica cardíaca, por meio da variabilidade da frequência cardíaca (VFC), têm se associado diretamente ao prognóstico cardiovascular^(^ ^4^ ^)^. Assim, quanto menor a VFC, maior é a chance de ocorrerem eventos coronarianos^(^ ^5^ ^)^. 

Além disso, alterações no controle autonômico cardíaco são descritas como características fisiopatológicas da obesidade infantil^(^ ^6^ ^-^ ^8^ ^)^. Em crianças e adolescentes obesos, observou-se redução na VFC e na modulação vagal e aumento no balanço simpato-vagal^(^ ^6^ ^,^ ^7^ ^)^. Essas informações sugerem, pelo menos em parte, que a disfunção autonômica cardíaca em crianças e adolescentes obesos relaciona-se à hiperativação simpática em detrimento da vagal. 

Contudo, na maioria dos estudos que avaliaram a VFC, as crianças e os adolescentes obesos apresentavam níveis pressóricos significativamente maiores quando comparados a seus pares não obesos^(^ ^6^ ^,^ ^8^ ^)^. Tal característica hemodinâmica poderia ser causa independente para a disfunção autonômica cardíaca observada nessa população. De fato, existe associação direta entre maiores níveis de pressão arterial e piora na modulação autonômica cardíaca^(^ ^9^ ^,^ ^10^ ^)^. Dessa forma, para excluir o efeito da elevação da pressão arterial na modulação autonômica cardíaca, o presente estudo teve como objetivo testar a hipótese de que crianças e adolescentes obesos normotensos apresentam disfunção autonômica cardíaca quando comparados a indivíduos não obesos também normotensos.

## Método

Pelo cálculo amostral baseado em artigo publicado anteriormente^(^ ^6^ ^)^, utilizando poder de 90% e erro α de 5%, constatou-se que deveriam ser recrutados no mínimo 23 indivíduos em cada grupo. Portanto, selecionaram-se 31 crianças e adolescentes obesos normotensos recrutados no Ambulatório de Obesidade e Hipertensão Infantil da Fundação Imepen de Juiz de Fora, MG, e 35 indivíduos controles não obesos recrutados na comunidade local. Todos os voluntários possuíam idade de oito e 17 anos, eram normotensos e não estavam sob uso de qualquer tipo de medicação. 

Explicaram-se todos os procedimentos da pesquisa aos representantes legais e aos voluntários e, após a concordância de ambos, o termo de consentimento livre e esclarecido foi assinado. Esta pesquisa foi aprovada pelo Comitê de Ética em Pesquisa da Universidade Federal de Juiz de Fora (UFJF) e desenvolvida no Hospital Universitário e na Faculdade de Educação Física e Desportos da UFJF.

Como os critérios de inclusão eram obesidade ou não e normotensão, utilizaram-se os seguintes procedimentos para definir os participantes: o peso corporal e a estatura foram mensuradas por meio de balança com estadiômetro (Filizola^(r))^ para o cálculo do índice de massa corpórea (IMC). Calculou-se o IMC dividindo-se o peso corporal em quilogramas pela altura em metros, ao quadrado. A obesidade foi definida como IMC maior que o percentil 95, conforme a idade e o sexo^(^ ^11^ ^)^. Mediu-se a pressão arterial no membro superior após 15 minutos em repouso na posição supina, de forma não invasiva, pelo método auscultatório, utilizando-se esfigmomanômetro de coluna de mercúrio (Takaoka^(r))^, em dois dias distintos, anteriores ao protocolo experimental. Aplicaram-se tamanhos adequados de manguito, considerando-se a circunferência do braço, segundo as recomendações da V Diretrizes Brasileiras de Hipertensão Arterial^(^ ^12^ ^)^. Classificaram-se como normotensos os indivíduos que apresentaram a média das duas medidas da pressão arterial sistólica e diastólica inferior ao percentil 90 conforme a idade e o sexo^(^ ^12^ ^)^, sendo inferior a 120mmHg para a pressão sistólica e a 80mmHg para a diastólica. Excluíram-se os indivíduos que estavam sob uso de qualquer medicação e/ou possuíam diagnóstico médico de doença metabólica, cardiovascular ou hormonal, além da obesidade. Verificaram-se esses critérios durante consulta clínica realizada pela responsável do serviço ambulatorial.

Avaliou-se a atividade física por meio do Questionário Atividade Física Habitual, validado na população juvenil brasileira, que quantifica os minutos dedicados à prática de atividade física habitual no período de 12 meses anteriores à sua aplicação^(^ ^13^ ^)^.

Durante a investigação, aferiu-se a pressão arterial não invasiva automaticamente pelo método oscilométrico (Dixtal, DX 2020), com o manguito posicionado no membro superior direito do voluntário. Registrou-se a frequência cardíaca continuamente, na posição supina, por meio de cardiofrequencímetro (Polar^(r)^, S810i). Para avaliar o controle autonômico cardíaco, os valores pontuais dos intervalos entre cada batimento cardíaco (iRR) coletados foram direcionados ao microcomputador pela transmissão de dados do receptor de pulso para o software *Polar Precision Performance**^(r)^*, utilizando-se interface de emissão de sinais infravermelhos. Todos os sinais incluídos apresentaram no máximo 3% de erro. Tais dados foram transferidos para o aplicativo Matlab, versão 6.0, para seleção automática dos cinco minutos de menor variância, por meio de rotina previamente implementada^(^ ^14^ ^,^ ^15^ ^)^. Realizou-se essa seleção automaticamente pela aplicação de janela móvel e o algoritmo matemático considerou apenas o trecho de cinco minutos ininterruptos do tacograma que apresentou a menor variância. Os trechos selecionados foram submetidos à análise visual e, somente na ausência de irregularidades marcantes nos intervalos R-R, foram considerados para a análise da VFC. As séries temporais com duração de cinco minutos selecionadas foram transferidas para o *software Kubios HRV Analysis*, versão 2.0^(^ ^15^ ^)^. Nesse aplicativo, corrigiram-se os artefatos, com filtro de nível médio do *software* e calcularam-se os índices de VFC, no domínio do tempo, com os valores dos intervalos R-R médios (MNN), em milissegundos (ms), que representa o inverso da frequência cardíaca; os valores dos desvios padrão dos intervalos R-R normais (SDNN), em ms, que reflete a VFC; a raiz quadrada da média dos quadrados das diferenças entre os intervalos R-R normais (RMSSD), em ms, e a porcentagem de intervalos R-R adjacentes com diferença superior a 50ms (pNN50), que refletem a modulação vagal cardíaca. Para estimar a função densidade espectral de potência pelo método não paramétrico da Transformada Rápida de Fourier^(^ ^16^ ^)^, removeu-se o componente de tendência da série temporal pelo método de suavização *a priori**^(^* ^17^ ^)^, após a interpolação por meio da *spline* cúbica, a uma frequência de 4Hz. Para a análise espectral da VFC, consideraram-se as bandas do espectro de potência de baixa frequência (LF - 0,04 a 0,15Hz), que refletem a modulação predominantemente simpática, e de alta frequência (HF - 0,15 a 0,4Hz), que refletem a modulação vagal cardíaca, expressas em potência absoluta (milissegundo ao quadrado - ms^2)^ e em unidades normalizadas (u.n.), além da razão LF/HF, que reflete o balanço simpato-vagal.

Avaliaram-se todos os voluntários no período da tarde para evitar a influência do ciclo circadiano. Inicialmente, realizaram-se as medidas antropométricas e, após 15 minutos de repouso na posição supina, aferiu-se a pressão arterial. Em seguida, registraram-se dez minutos contínuos de frequ*ência cardíaca para *posterior cálculo da VFC.

Os dados foram expressos como média±desvio padrão. Verificou-se a normalidade dos dados pelo teste de Kolmogorov-Smirnov. As possíveis diferenças entre os grupos para variáveis com distribuição normal foram analisadas pelo teste *t* de Student para amostras independentes e, para as variáveis que não apresentaram distribuição normal, pelo teste U de Mann-Whitney. Realizou-se a análise de covariância (ANCOVA) para controlar os possíveis efeitos da idade e do sexo. Para todos os testes estatísticos, considerou-se diferença significativa quando *p*<0,05 e utilizou-se o programa *Statistica*, versão 8.0 (Statsoft, USA).

## Resultados

Os resultados antropométricos, hemodinâmicos e de prática de atividade física habitual são apresentados na Tabela 1. Os grupos foram semelhantes quanto a idade, nível habitual de atividade física e, como esperado, não houve diferenças significativas nos níveis de pressão arterial sistólica e diastólica. Além disso, os níveis pressóricos encontravam-se dentro da faixa considerada ótima. 

**Tabela 1:** Características antropométricas, hemodinâmicas de repouso e da atividade física semanal dos grupos Obeso e Não Obeso

Os resultados da VFC no domínio do tempo e da frequência são mostrados na Tabela 2. Os índices MNN, SDNN e RMSSD foram semelhantes entre os grupos e o índice pNN50 foi menor no Grupo Obeso, comparado ao Grupo Não Obeso. Quanto à VFC no domínio da frequência, as bandas espectrais LF e HF, em potência absoluta, foram semelhantes entre os grupos. Por outro lado, a banda HF (u.n.) foi significativamente menor e as variáveis LF (u.n.) e LF/HF foram maiores no Grupo Obeso. Entre as variáveis utilizadas para ANCOVA, observou-se que a obesidade foi fator independente para os baixos valores do pNN50 e da banda HF (u.n.) e para os altos valores da banda LF (u.n.) e da razão LF/HF do Grupo Obeso (Tabela 3).

**Tabela 2:** Variabilidade da frequência cardíaca no domínio do tempo e da frequência entre os grupos Obeso e Não Obeso

**Tabela 3:** Análise de covariância sobre a variabilidade da frequência cardíaca

## Discussão

O principal achado do presente estudo é que crianças e adolescentes obesos normotensos apresentam comprometimento da modulação autonômica cardíaca, quando comparados a seus pares não obesos também normotensos. O índice pNN50 e a banda HF (u.n.), que indicam a modulação vagal cardíaca, foram menores nos indivíduos obesos. Além disso, a banda LF (u.n.), que reflete a modulação predominantemente simpática cardíaca, e a razão LF/HF, indicativa do balanço simpato-vagal, mostraram-se elevadas no Grupo Obeso. Todos os resultados foram independentes dos efeitos da idade e do gênero. 

A associação positiva entre obesidade e níveis pressóricos elevados tem sido relatada consistentemente^(^ ^18^ ^)^, sendo que essas alterações hemodinâmicas são explicadas principalmente pela hiperatividade do sistema nervoso simpático^(^ ^19^ ^)^. Portanto, há claras evidências de que o ganho excessivo de peso corporal pode provocar alterações hemodinâmicas e deficiência no controle autonômico^(^ ^19^ ^,^ ^20^ ^)^. Nesse sentido, Guízar *et al**^(^* ^8^ ^)^ verificaram que adolescentes obesos possuem comprometimento significativo do balanço simpato-vagal, representado por elevação da razão LF/HF. Porém, neste trabalho, os adolescentes, apesar de normotensos, apresentavam níveis pressóricos significativamente maiores que seus pares não obesos. Neste estudo, também houve alteração negativa do balanço simpato-vagal por meio de maiores valores da razão LF/HF nas crianças e nos adolescentes obesos. No entanto, diferentemente do trabalho de Guízar *et al**^(^* ^8^ ^)^, no qual os níveis pressóricos já estavam elevados, nossos resultados mostram que a obesidade, por si só, foi responsável por prejudicar a modulação autonômica cardíaca de crianças e adolescentes obesos independentemente dos valores pressóricos de repouso. Isso porque os valores de pressão arterial, além de classificarem os indivíduos investigados como normotensos, foram semelhantes entre o Grupo Obeso e Não Obeso. 

Não há dúvidas de que o prejuízo do controle autonômico cardíaco adicionalmente à elevação da pressão arterial esteja presente na obesidade. No trabalho de Riva *et al**^(^* ^6^ ^)^, além do balanço simpato-vagal comprometido, os adolescentes obesos também apresentaram modulação vagal significativamente reduzida, representada por menores valores do índice pNN50. Dessa forma, ao se considerarem os achados desses pesquisadores^(^ ^6^ ^)^ juntamente com os resultados do presente estudo, verifica-se que a disfunção autonômica observada na obesidade pode se caracterizar por ativação simpática em detrimento da atuação vagal. Concordando com tais achados, Kauffman *et al**^(21) ^*verificaram, em adolescentes obesos, que a banda HF em u.n. relacionada à modulação vagal estava reduzida, enquanto a banda LF em u.n. e a razão LF/HF estavam aumentadas. Além disso, esses autores notaram que o prejuízo no controle autonômico cardíaco na obesidade infantil associa-se aos níveis de leptina, à resistência insulínica, ao aumento do estresse oxidativo e da inflamação, sendo que essa relação é mediada primariamente pelo tecido adiposo. 

A razão LF/HF tem sido proposta por ser uma medida acurada do balanço simpato-vagal sobre o coração. Assim, valores elevados dessa razão podem indicar maior modulação simpática sobre o sistema cardiovascular^(^ ^22^ ^,^ ^23^ ^)^. De fato, no presente estudo, o Grupo Obeso normotenso apresentou maior valor da razão LF/HF, comparado ao Grupo Não Obeso, o que indica que o tecido adiposo é um dos responsáveis pelo aumento no estímulo simpático, embora outros mecanismos também possam estar relacionados. Confirmando a representação fisiológica de LF/HF, as componentes espectrais das bandas LF e HF, em u.n., revelam a atuação balanceada dos dois ramos do sistema nervoso autônomo no controle dos batimentos cardíacos^(^ ^22^ ^)^. A existência de relação linear entre as mudanças da banda LF (u.n.) e da frequência cardíaca durante testes incrementais de manobra postural ortostática já foi demonstrada, reforçando a ideia de que acréscimos nos valores espectrais desse índice indicam possível aumento da atuação simpática sobre o coração^(^ ^24^ ^)^. Dessa forma, mais uma vez, nossos resultados confirmaram o predomínio da modulação simpática cardíaca no Grupo Obeso, verificado pelos valores significativamente elevados de LF (u.n) desses indivíduos quando comparados aos não obesos. Consequentemente, no presente estudo, observou-se o prejuízo da atuação vagal cardíaca nos indivíduos obesos por meio da HF (u.n.) reduzida. 

Apesar de não ter sido objeto de estudo neste trabalho, alguns mecanismos neuro-humorais podem explicar a disfunção autonômica cardíaca observada na obesidade. Dentre eles, merecem destaque elevações nos níveis de insulina, leptina, citocinas pró-inflamatória, estresse oxidativo e catecolaminas^(^ ^25^ ^,^ ^26^ ^)^. Tais mecanismos são responsáveis pela hiperativação simpática observada nos indivíduos obesos^(19) ^e, dessa forma, é possível especular que as crianças e os adolescentes investigados também já possam apresentar alterações nesse sentido. 

A detecção de disfunção autonômica em crianças e adolescentes ressalta a presença de maior risco cardiovascular associado à obesidade infantil e, principalmente, às consequências de aumento da morbimortalidade na vida adulta^(^ ^1^ ^)^. Esses resultados alertam sobre a necessidade de ações que busquem a atuação precoce e evitem a evolução para complicações cardiovasculares. Programas de educação em saúde, além de medidas intervencionistas, como mudanças no estilo de vida que envolvam a adoção de dieta equilibrada e prática de atividade física regular, devem ser executados por equipes multiprofissionais para prevenir e tratar a obesidade infantil. 

Uma limitação do presente estudo é o fato de não ter sido realizada alguma medida do estado maturacional dessas crianças e adolescentes. Com o conhecimento de que o estado maturacional é influenciado por idade e sexo, conduziu-se a ANCOVA para controlar os possíveis efeitos dessas variáveis. Nossos resultados mostraram que as crianças e adolescentes obesos apresentaram o índice pNN50 e a banda HF em u.n. reduzidos e a banda LF em u.n. e a razão LF/HF aumentadas independentemente da idade e do sexo. Portanto, mesmo que o nível maturacional não tenha sido avaliado, é improvável que esse fator esteja influenciando nossos resultados.

Pode-se concluir que crianças e adolescentes obesos, mesmo normotensos, apresentam disfunção do controle autonômico cardíaco quando comparados a seus pares não obesos também normotensos.
